# Integration of Transcriptome and Metabolome Reveals the Formation Mechanism of Red Stem in *Prunus mume*

**DOI:** 10.3389/fpls.2022.884883

**Published:** 2022-05-06

**Authors:** Like Qiu, Tangchun Zheng, Weichao Liu, Xiaokang Zhuo, Ping Li, Jia Wang, Tangren Cheng, Qixiang Zhang

**Affiliations:** Beijing Key Laboratory of Ornamental Plants Germplasm Innovation & Molecular Breeding, National Engineering Research Center for Floriculture, Beijing Laboratory of Urban and Rural Ecological Environment, Engineering Research Center of Landscape Environment of Ministry of Education, Key Laboratory of Genetics and Breeding in Forest Trees and Ornamental Plants of Ministry of Education, School of Landscape Architecture, Beijing Forestry University, Beijing, China

**Keywords:** *Prunus mume*, transcriptome, metabolome, flavonoid biosynthesis, anthocyanin biosynthesis, red stem, xylem color

## Abstract

*Prunus mume* var. purpurea, commonly known as “Red Bone”, is a special variety with pink or purple-red xylem. It is famous due to gorgeous petals and delightful aromas, playing important roles in urban landscaping. The regulation mechanism of color formation in *P. mume* var. purpurea stem development is unclear. Here, we conducted a comprehensive analysis of transcriptome and metabolome in WYY (‘Wuyuyu' accession, red stem) and FLE (‘Fei Lve' accession, green stem), and found a total of 256 differential metabolites. At least 14 anthocyanins were detected in WYY, wherein cyanidin 3,5-O-diglucoside and peonidin3-O-glucoside were significantly accumulated through LC-MS/MS analysis. Transcriptome data showed that the genes related to flavonoid-anthocyanin biosynthesis pathways were significantly enriched in WYY. The ratio of dihydroflavonol 4-reductase (*DFR*) and flavonol synthase (*FLS*) expression levels may affect metabolic balance in WYY, suggesting a vital role in xylem color formation. In addition, several transcription factors were up-regulated, which may be the key factors contributing to transcriptional changes in anthocyanin synthesis. Overall, the results provide a reference for further research on the molecular mechanism of xylem color regulation in *P. mume* and lay a theoretical foundation for cultivating new varieties.

## Introduction

Color formation is one of the main quality traits of ornamental plants. The most widespread non-green pigments in flowers, fruits and various organs are generally classified into three categories: anthocyanins, carotenoids, and betalains (Boldt et al., 2014). Anthocyanins, a class of secondary metabolites, are important water-soluble pigments that are widely accumulated in vascular plants and the different substituents on the B ring of flavonoid basic skeleton result in a variety of colors ranging from red to blue (Tanaka et al., 2008). Besides providing beautiful pigmentation in flowers to attract pollinators and seed spreaders, anthocyanins also play a key role in signal transmission between plants and microorganisms (Harborne and Williams, 2000), photoprotection during photosynthesis (Hughes et al., 2005), antioxidant activity (Wei et al., 2018), and UV protection (Jansen et al., 1998). Recent studies have indicated that anthocyanins can ameliorate drug-induced cognitive deficits (Jo et al., 2015) and anthocyanin-rich diets are associated with decreased cardiovascular diseases and mortality (Isaak et al., 2017). Therefore, on account of the functional diversity, anthocyanin have attracted much more attention, becoming a research hotspot in the field of secondary metabolism of horticultural crops.

So far, more than 700 compounds have been found in plants, which mainly derive from six anthocyanidins aglycones (Celli et al., 2018). Anthocyanin biosynthesis is rather conservative in plants. It occurs on the cytoplasmic surface of the endoplasmic reticulum. The biosynthetic pathway has been extensively studied in *Arabidopsis thaliana* (Martens et al., 2010), *Petunia hybrida* (Jonsson et al., 1984), *Zea Mays* (Harborne and Gavazzi, 1969) and *Dianthus* (Stich and Wurst, 1992). In the first step, phenylalanine is catalyzed by phenylalanine ammonia-lyase (PAL) to produce 4-coumaroyl-CoA and 3 malonyl-CoA. In the second stage, dihydroflavonols are generated from coumaroyl-CoA under the catalysis of chalcone synthase (CHS), chalcone isomerase (CHI) and flavanone 3-hydroxylase (F3H), which is a key step in the metabolism of flavonoids. Flavonoid 3′-hydroxylase (F3′H) and flavonoid 3′,5′-hydroxylase (F3′5′H) catalyze the hydroxylation of dihydrokaempferol to form dihydroquercetin and dihydromyricetin, and determine the hydroxylation pattern of flavonoid and anthocyanin B ring, which are important enzymes for the synthesis of cyanidin and delphinidin (Zhuang et al., 2019). Subsequently, under the action of dihydroflavonol 4-reductase (DFR), dihydroflavonols are reduced to the corresponding 3,4-cis-leucoanthocyanidins, which are then catalyzed by anthocyanidin synthase (ANS) to form colored anthocyanidins. Unstable colored anthocyanins are modified by glycosylation, methylation, and acetyltransferase to form different types of stable anthocyanin polymers that give plants different colors. The high expression level of *DFR, ANS* and *anthocyanidin 3-O-glucosyltransferase* (*UFGT*) genes in downstream of the anthocyanin biosynthetic pathway tends to promote flower or fruit coloring (Wang et al., 2017). The *UFGT* has been identified as a key gene for determining the concentration and accumulation of anthocyanins, causing red color petals in *Prunus mume* (Wu et al., 2017). Silencing of *FaDFR* in strawberry decreases the anthocyanin content, rendering pale fruit color (Lin et al., 2013).

In addition to the above-mentioned structural genes, transcription factors can activate or inhibit the temporal and spatial expression of structural genes through specific proteins, thus affecting the intensity and pattern of anthocyanin biosynthesis. Many studies have shown that the synthesis of anthocyanins is regulated by a protein complex formed by a MYB transcription factor, a basic helix-loop-helix protein (bHLH), and a WD-repeat protein, which bind to the promoters of structural genes to induce their transcription (Koes et al., 2005). In the model plant *Arabidopsis thaliana*, the AtTT2-AtTT8-AtTTG1 transcription complex can promote the expression of *DFR, ANS*, and *TT19* genes in the proanthocyanidin synthesis pathway (Xu et al., 2013) and AtPAP1-AtTT8/GL3-AtTTG1 can also actuate the expression of structural genes in the anthocyanin synthesis pathway (Tohge et al., 2005; Gonzalez et al., 2008). In petunia, PhAN11-PhAN1-PhAN2 regulates anthocyanin synthesis in petals by adjusting the expression of *DFR* and *CHS*, while AN11-AN1-AN4 regulates anther development (Quattrocchio et al., 2006). In *Gerbera hybrida, GMYB10* interacts with bHLH factor *GMYC1* to superintend anthocyanin synthesis in leaves, floral stems, and flowers by actuating the late gene *DFR* (Elomaa et al., 2003). The *GtMYB3* transcription factor and *GtbHLH1* work synergistically to affect the expression of the *F3'5'H* gene, which in turn promotes the enrichment of gentiodelphin in *Gentian trifloral* (Nakatsuka et al., 2008). The *MdMYB10* induces cyanidinin accumulation and slightly increases the expression of *DFR* in apples with overexpression of *MdMYB10*. Meanwhile, *MdMYB10* co-expressed with *MdbHLH3* and *MdbHLH33* promote the biosynthesis of anthocyanins (Espley et al., 2007).

*Prunus mume* (also named mei), a member of the Rosaceae family, is a traditional flower species in China that has high ornamental value due to its colorful corolla, wispy fragrance, and varying flower types. With the rapid development of high-throughput sequencing technology, the whole genome sequencing of *P. mume* was completed in 2012, which provided important data support for revealing the biological characteristics of bud dormancy, floral scent, and plant architecture. However, there are few systematic studies on the formation mechanism of wood color in *P. mume*, and the metabolic pathway and molecular regulatory mechanism of wood color are still unclear. Transcriptome and metabolome are combined to identify and analyze the interaction of single and multiple genes in metabolic pathways in many plants, including potato (Cho et al., 2016), grape hyacinth (Lou et al., 2014), turnip (Zhuang et al., 2019), and crape myrtle (Qiao et al., 2019), providing a powerful tool to analyze the mechanisms of plant tissue-specific metabolism and secondary metabolism. Combined transcriptome and metabolome studies can not only detect the abundance of transcripts, but also provide a new perception of the flow of metabolism.

Here, we used ultra-performance liquid chromatography and mass spectrometry to survey the differences of metabolites conferring the red stems of *P. mume* cultivar ‘Wuyuyu' compared to ‘Fei Lve', which feature red and green stems, respectively. Besides, we employed RNA-seq technique to identify the key candidate genes involved in anthocyanin metabolism of differential pigmentation and then verified by quantitative real-time polymerase chain (qRT-PCR). The outcomes of the study may provide valuable information for understanding the molecular mechanism of the wood color formation at the transcriptomic and metabolomic levels in *P. mume*.

## Results

### Metabolome Profiling of LC-MS/MS Data

**Figure 1:**
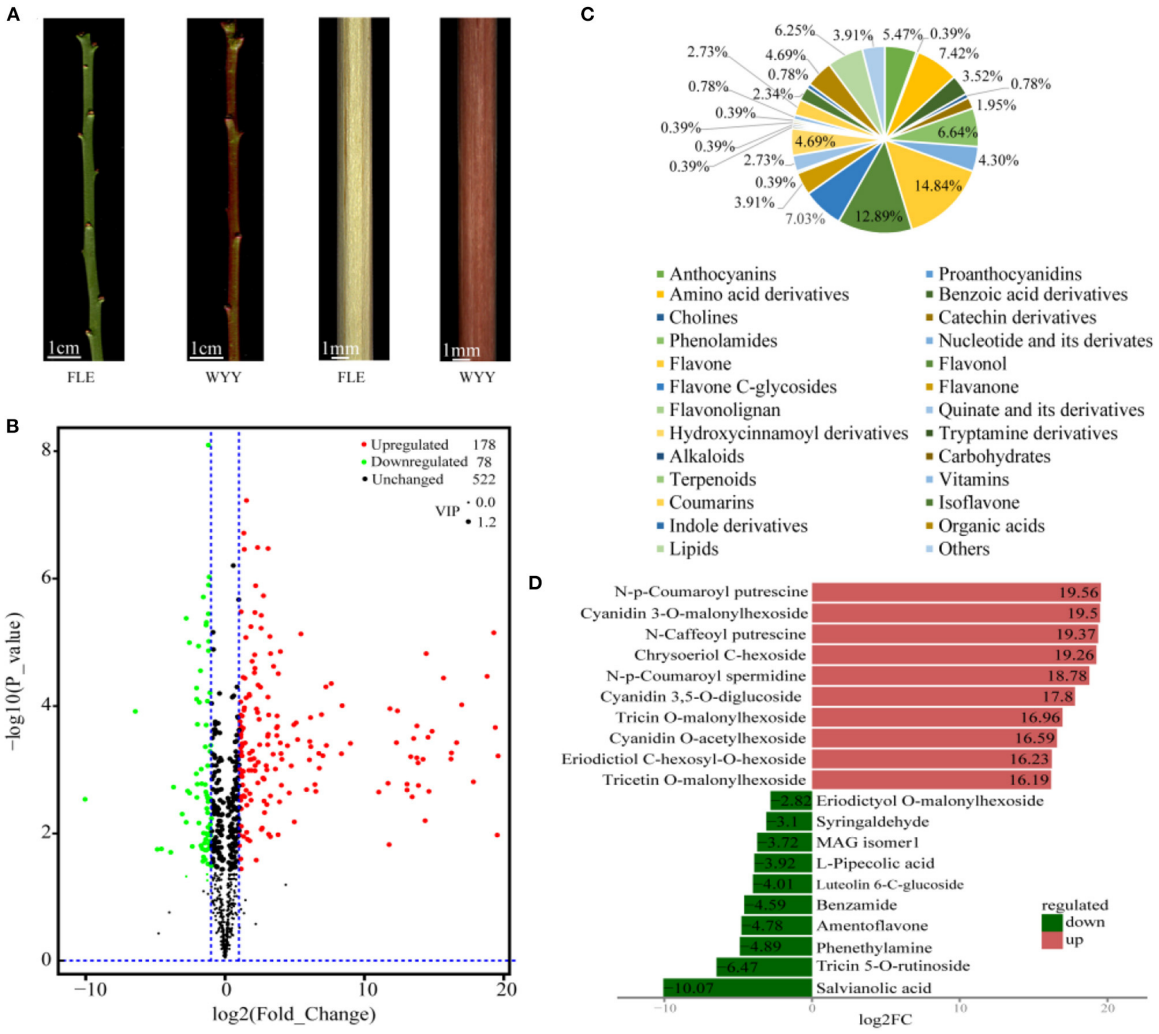
Morphological characterization and differentially accumulating metabolites between WYY and FLE. **(A)** Green-stem cultivar ‘Fei Lve' (FLE) and red-stem cultivar ‘Wuyuyu' (WYY). **(B)** Volcano plot of the 778 metabolites identified. Differential metabolites were defined with fold change ≥ 2 or ≤ 0. 5, and VIP ≥ 1. **(C)** Pie chart depicting the biochemical categories of the differential metabolites identified between WYY and FLE. **(D)** The top 10 significantly differentially expressed metabolites.

Two *P. mume* cultivars with different stem colors [‘Fei Lve' (FLE) and ‘Wuyuyu' (WYY)] were chosen for this study (Figure 1A). To evaluate the components of FLE and WYY, widely-targeted metabolomics was used to analyze the metabolic profiles based on UPLC-MS/MS. A total of 778 metabolites grouped into 26 classes were identified from FLE and WYY samples and the most abundant metabolites were the flavonoids (Supplementary Table S1).

Principal component analysis (PCA) revealed the overall metabolic differences between WYY and FLE samples. It can be clearly seen that the two groups were separated into distinct clusters on the PC1 × PC2 score plot, where the variances of PC1 and PC2 were 83.11%, 5.23%, respectively (Supplementary Figure S1). Multivariate statistics of the metabolite concentration data was performed to access the differences in sample accumulation patterns. These two groups could be easily distinguished from each other on the heatmap, indicating significant biochemical differences in metabolites between WYY and FLE (Supplementary Figure S2).

### Differentially Accumulated Metabolite Analysis Based on OPLS-DA

PCA is insensitive to the variables with small correlation, while the orthogonal projections to latent structures-discriminant analysis (OPLS-DA) can maximize the discrimination between groups, which is conducive to searching for differential metabolites. We used OPLS-DA models to filter differential compounds between two groups of samples by removing irrelevant differences. As described in materials and methods, the variable importance in projection (VIP) values and the *P*-values of univariate statistical *t*-test were used to screen the metabolites with significant differences. Furthermore, the high R2 and Q2 values indicated that the multivariate model had good quality and predictive ability (Supplementary Figure S3). Overall, we putatively identified 256 differentially accumulated metabolites (DAMs), including 78 down-regulated and 178 up-regulated metabolites in WYY compared with FLE (Figure 1B and Supplementary Table S2). The DAMs can be divided into more than 20 different categories, and flavonols, amino acids, amino acid derivatives, lipids, and anthocyanins were significantly different between the two cultivars (Figure 1C). The most up-regulated metabolites were N-p-Coumaroyl putrescine, followed by cyanidin 3-O-malonylhexoside and N-caffeoyl putrescine. The top three down-regulated metabolites were salvianolic acid, tricin 5-O-rutinoside, and phenethylamine (Figure 1D).

To further understand the biological classification and pathways of these metabolites, the DAMs were assigned to KEGG database for enrichment analysis. Notably, the relative abundance of differential metabolites between FLE and WYY was mainly related to metabolic and secondary metabolites biosynthesis, including phenylpropanoid biosynthesis, flavonoid biosynthesis and anthocyanin biosynthesis (Supplementary Figure S4). Flavonoids occupied a large proportion in the composition of plant pigments.

### Metabolites in the Anthocyanin Biosynthetic Pathway

Anthocyanins are water-soluble pigments existing in the vacuoles of cells that absorb different wavelengths of light and exhibit different colors. Twenty-four anthocyanins were identified in all samples, among which 14 were differentially expressed (Table 1). In general, the content and species of anthocyanins in the stems of WYY were higher than those of FLE. Quantitative profiles showed that cyanidin 3-O-malonylhexoside, cyanidin 3,5-O-diglucoside, cyanidin O-acetylhexoside, pelargonidin 3-O-malonylhexoside, pelargonidin O-acetylhexoside, which are the main source of the reddish-purple color in the plant kingdom, were only detected in WYY and their contents were significantly higher than FLE, while the contents of the other nine anthocyanins varied between the two cultivars. Anthocyanins of the same species were detected in the two cultivars, but their expression levels varied significantly. Notably, the level of peonidin in FLE was slightly higher than that in WYY, suggesting anthocyanin accumulation in the green stem.

**Table 1:** Differentially expressed anthocyanins in WYY and FLE.

**Metabolite name**	**Ion**	**Ion**	**log_**2**_FC**	**VIP**
	**abundance**	**abundance**		

	**FLE**	**WYY**	**WYY/FLE**	
Cyanidin 3-O-malonylhexoside	N/A	6.68E+06	19.50	1.20
Cyanidin 3,5-O-diglucoside	N/A	2.05E+06	17.80	1.28
Cyanidin O-acetylhexoside	N/A	8.88E+05	16.59	1.30
Pelargonidin 3-O-malonylhexoside	N/A	9.04E+04	13.29	1.29
Pelargonidin O-acetylhexoside	N/A	1.84E+04	11.00	1.27
Peonidin O-hexoside	3.81E+05	1.27E+08	8.37	1.31
Cyanidin O-syringic acid	1.93E+05	6.16E+07	8.32	1.29
Cyanidin 3-O-rutinoside	3.56E+05	6.90E+07	7.60	1.31
Pelargonidin 3-O-beta-D-glucoside	4.42E+05	6.53E+07	7.21	1.31
Cyanidin 3-O-glucoside	7.72E+04	9.81E+06	6.99	1.29
Delphinidin	1.43E+04	9.50E+05	6.05	1.29
Delphinidin 3-O-rutinoside	2.41E+03	7.40E+04	4.94	1.21
Malvidin 3,5-diglucoside	5.09E+04	1.11E+05	1.12	1.04
Peonidin	6.72E+05	2.06E+05	−1.70	1.26

### HPLC-MS Analysis of Pigmentation in Stems

To further confirm the role of anthocyanins in stem pigmentation, the contents of anthocyanins in several *P. mume* cultivars were determined. In addition to WYY and FLE, we selected two other cultivars, ‘Fenhong Zhusha' (FHZS, red stem) and ‘Zaohua Lve' (ZHLE, green stem) for quantitative determination. Anthocyanins in the red and green samples were identified and relatively quantified by HPLC-MS based on retention time and mass spectrometry. The content of anthocyanins in samples was calculated by the percentage of peak area between sample solution and standard solution.

**Figure 2:**
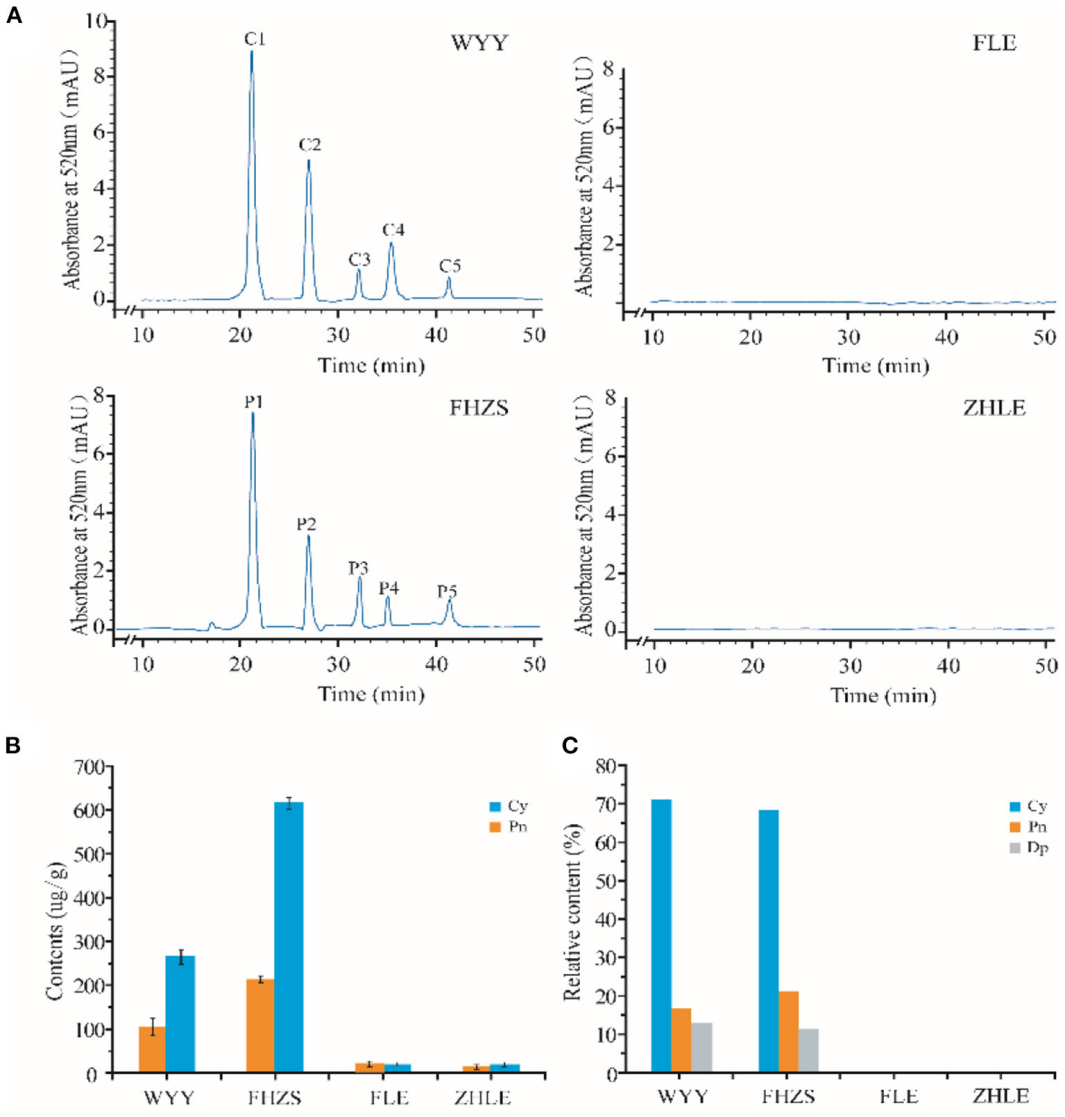
HPLC-MS analysis of pigmentation in stems. **(A)** Results of anthocyanins composition in mei cultivars with different stem colors by HPLC-MS analysis. **(B)** The absolute quantification of anthocyanins content in four mei cultivars. **(C)** The relative quantification of anthocyanins content in four mei cultivars. WYY and FHZS were red stem cultivars, FLE, and ZHLE were green stem cultivars.

HPLC showed that the maximum absorption wavelength of WYY and FHZS was 520 nm, which is the characteristic peak of anthocyanins pigments that is different from other flavonoid compounds, while FLE and ZHLE did not detect the specific peak of anthocyanins. Since WYY and FHZS belong to the same cultivar group, we found the same anthocyanin species between them, but the amount of anthocyanins varied greatly. There were five obvious peaks in WYY extractions with retention times of 21.62, 27.72, 31.97, 36.31, and 41.49 min, respectively (Figure 2A). These peaks were further identified by mass spectrometry (Table 2). Quantification of these peaks indicated that both cultivars contained three typical red-purple anthocyanin compounds: 70.82% for Cy, 16.47% for Pn and 12.71% for Dp in WYY; and 68.39% for Cy, 20.89% for Pn and 10.72% for Dp in FHZS (Figure 2C). This shows that Cy3G5G and Cy3Ru are the most important anthocyanins in the red stem formation of WYY and FHZS. Since anthocyanins were detected in FLE in the metabolome, the absolute contents of Cy and Pn in the four cultivars were tested to verify the correctness of the metabolome data (Figure 2B). Between red stem cultivars, FHZS had the highest anthocyanin accumulation level, which was 615 μg/g of fresh weight (FW). However, the content in WYY (265 μg/g of FW) was much higher than FLE (1.29 μg/g of FW). Only trace amounts of anthocyanins were detected in FLE and ZHLE, indicating that different statistical methods may produce certain errors in the results under the condition of too low content (Figure 2B).

**Table 2:** Structure identification of anthocyanidins in WYY.

	**Ingredient**	**Retention time (min)**	**Molecular ion (*m*/*z*)**	**MS^**2**^ (*m*/*z*)**	**Annotation**
**WYY**	C1	21.62	949, 757, 595, 449, 287	287.1	Cyanidin 3-O-rutinoside
	C2	27.72	611, 449, 287,	287.1	Cyanidin 3,5-diglucoside
	C3	31.97	465, 303	303.1	Delphinidin 3-O-glucoside
	C4	36.31	463, 301	301.7	Peonidin 3-O-glucoside
	C5	41.49	609, 463, 447, 301	301.7	Peonidin 3-O-rutinoside

### Global Analysis of RNA-seq Data

**Figure 3:**
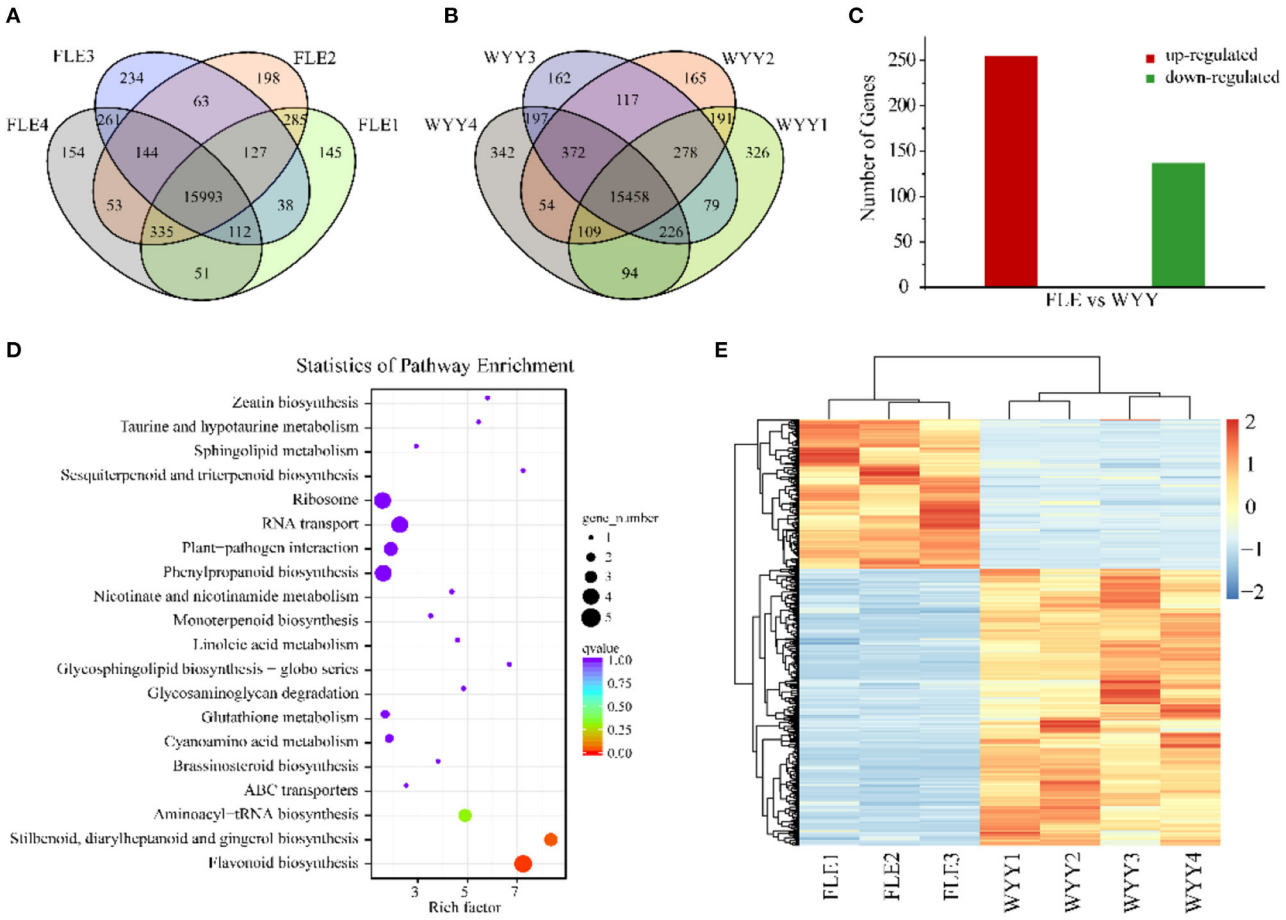
Transcriptome profiles of WYY and FLE. **(A)** Venn diagram depicting the shared and specific unigenes among the four replicates of the FLE group. **(B)** Venn diagram depicting the shared and specific unigenes among the four replicates of WYY group. **(C)** The number of up-regulated and down-regulated genes. **(D)** KEGG functional analysis of DEGs. The X-axis represents the enrichment factor. The Y-axis represents the pathway. The color indicates the *q*-value (high: red, low: blue). A lower *q*-value indicates a more significant enrichment. Point size indicates the DEG number (the larger dots refer to a larger amount). **(E)** Hierarchical clustering of DEGs expression.

To investigate the difference of gene expression level in anthocyanin metabolism of WYY, total RNA isolated from the stems of FLE and WYY were sequenced for transcriptomic analysis, which is consistent with samples used for metabolomic analysis. A total of 61.01 Gb clean data was generated with average 6.52 Gb per sample, and the percentage of Q30 base rates was more than 92.86% and the rate of total mapping ranged from 85.88% to 91.19%. A total of 15,933 and 15,458 core genes were found in the FLE and WYY groups, respectively (Figures 3A,B and Supplementary Table S3). The PC1 displays the distinct separation between red-colored and green-colored samples, which is consistent with the results of the metabolome. All the assembled unigene sequences were aligned to the public databases using the BLAST program for gene function annotations and protein prediction. Finally, 1,631 novel genes were discovered, of which 1,424 had functional annotations.

### GO and KEGG Analysis of DEGs

To identify alterations in gene expression levels, differential gene screening was performed based on a false discovery rate (FDR) ≤ 0.01 and |log2 (foldchange)|≥1. The Pearson's correlation coefficient analysis was used to evaluate the reproducibility of the differential gene expression library. The FLE4 (T08) library had a poor correlation with others (Supplementary Figure S5). To improve the repeatability between samples, the T08 library was removed and the heatmap clustering analysis was performed again (Figure 3E). Finally, a total of 392 DEGs including 255 up-regulated genes and 137 down-regualted genes were obtained from WYY/FLE libraries (Figure 3C and Supplementary Table S4). Functional annotation analyses revealed that 385 (98.21%), 150 (38.26%), 95 (24.23%), 189 (48.21%), 311 (79.34%), 283 (72.19%), and 117 (29.84%) unigenes were significantly enriched in the NR, COG, GO, KOG, PFAM, Swiss-Prot and KEGG databases, respectively. We obtained about 95 genes, which were classified into three main categories, and then grouped them into 44 sub-categories according to the GO classification, namely biological processes, molecular functions and cellular component (Supplementary Figure S6). In the biological process, GO terms were mainly enriched in the metabolic and the cellular processes, followed by the single-organism process. For the cellular component category, DEGs associated with cell and cell part were the most abundant. Within molecular function, the main sub-categories were catalytic activity and binding, followed by molecular function regulator, indicating that transcription factors and high enzymatic activity are closely related to the regulation of WYY stem coloration.

We then compared the DEGs against the KEGG pathways to obtain significantly enriched pathways. A total of 117 DEGs were mapped into 43 pathways, while only 4 pathways were significantly enriched. The top 20 enriched pathways were used to draw the enrichment map (Figure 3D). The most represented pathways comprised of one flavonoid biosynthesis pathway (ko00941), one phenylpropanoid biosynthesis pathway (ko00940), followed by stilbenoid, diarylheptanoid and gingerol biosynthesis (ko00945) and RNA transport (ko03013), which can form a metabolic network. Basically, naringenin produced by the “phenylpropanoid biosynthesis” pathway is used by the “flavonoid biosynthesis pathway” to produce dihydroflavonols. Then, the flavonoid metabolic pathway produces leuco-anthocyanins as the substrate of the anthocyanin biosynthesis pathway, which is modified to produce various types of anthocyanins and transported to the vacuole for stable existence.

### DEGs Related to Color Development

**Figure 4:**
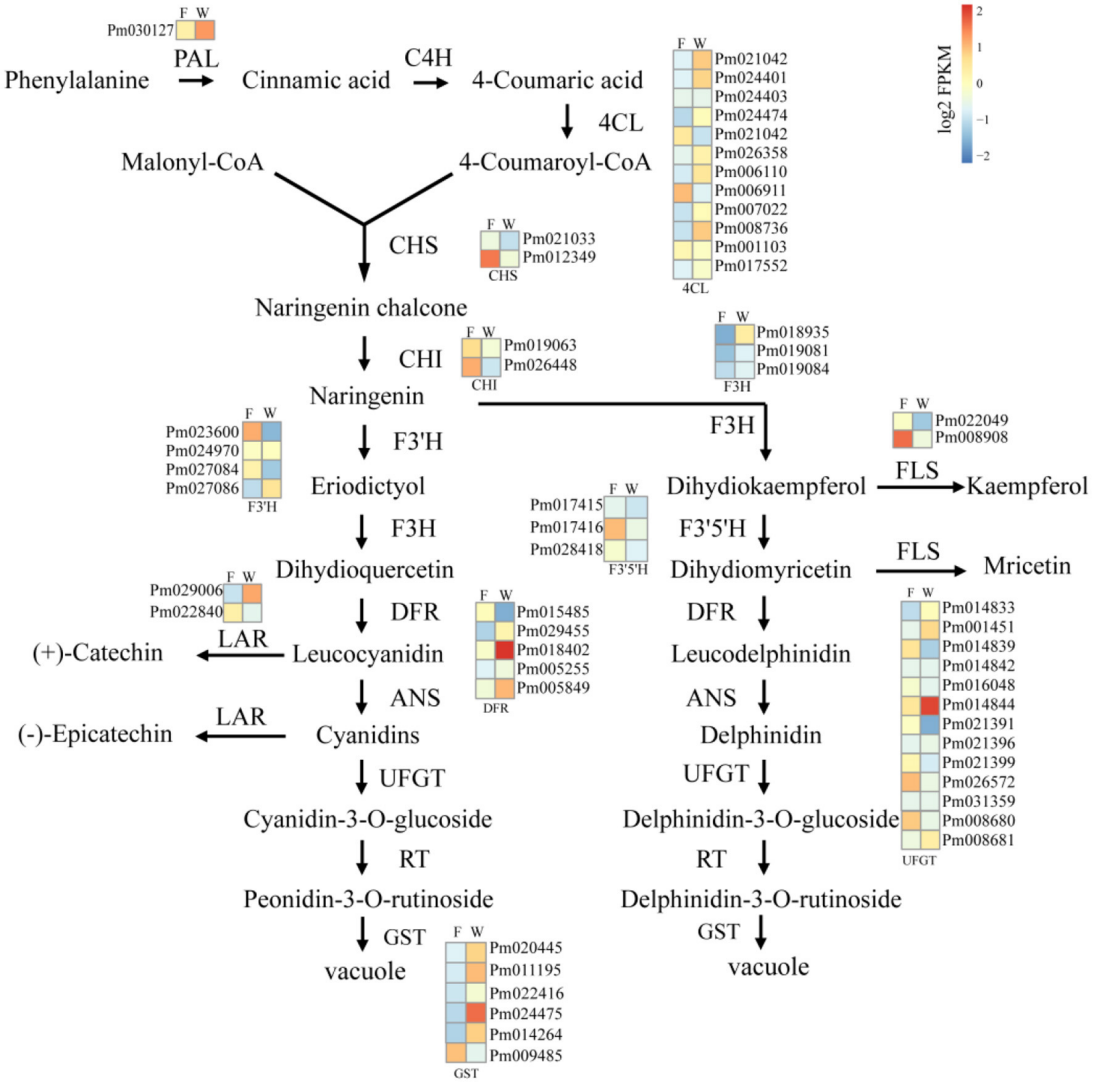
The analysis of unigenes involved in anthocyanin biosynthesis of WYY. Phenylalanine ammonia-lyase (PAL), cinnamic acid 4-hydroxylase (C4H), 4 coumarate-CoA ligase (4CL), chalcone synthase (CHS), chalcone isomerase (CHI), flavanone 3-hydroxylase (F3H), flavonol synthase (FLS), anthocyanidin 3-O-glucosyltransferase (UFGT), Dihydroflavonol 4-reductase (DFR), anthocyanidin reductase (ANR), leucoanthocyanidin dioxygenase (LAR). F:FLE, W:WYY. Squares represent different transcripts of genes. In the heat maps, blue-yellow-red scale represent low and high transcript expression, respectively.

Considering the differences between WYY and FLE in the major anthocyanin types, and the KEGG enrichment showed that DEGs were enriched in the flavonoid metabolic pathway, 55 genes involved in anthocyanin biosynthesis were investigated, including *PAL, 4CL, CHS, F3'5'H, F3'H, DFR, LDOX, UFGT*, and *GST*. Genes encoding anthocyanin synthesis, modification and transport (*DFR, UFGT* and *GST*) showed higher expression levels in WYY, implying that these genes may be vital for the accumulation of anthocyanins. In contrast, *chalcone synthases* (*CHS*), *coumaricacid3-hydroxylase* (*C3H*), and *flavonol synthase* (*FLS*) were down-regulated in WYY compared with FLE (Figure 4). Two homologous genes of hydroxycinnamoyl-CoA shikimate/quinate *hydroxycinnamoyl transferases* (*HCT*) showed opposite expression patterns, one was up-regulated and the other was down-regulated.

### DEGs Related to Flavonoid Transport

Anthocyanin glycosides are usually transported into the vacuole *via* transporters for storage or isolation. We searched all these transcripts encoding proteins implicated in transport and catabolism pathways in the functional annotations. The results showed that 27 key unigenes were differentially expressed, of which 18 were up-regulated and 9 were down-regulated. Among the 27 genes, cytochrome P450 families, ABC transporter family, and glutathione S-transferase-like protein had the most members (Figure 4, Supplementary Table S5). The expression levels of four cytochrome P450 genes were up-regulated and four genes were down-regulated in WYY. Interestingly, three glutathione S-transferase were expressed in all four WYY samples, but not in any of the FLE samples. Moreover, a multidrug and toxic efflux transporter protein (TT12) was significantly up-regulated in WYY.

### DEGs Related to Sugar and Hormones Metabolism

Among the 12 putative functional homologous genes implicated in hormone and sugar metabolism, homologs of *SAUR-like auxin-responsive protein* (*SAUR32*), *auxin efflux carrier component 5* (*PIN5*), and *brassinosteroid insensitive 1* (*BI1*), and *cytokinin dehydrogenase 3* (*CKX3*) were significantly up-regulated in WYY. Three transcripts were significantly down-regulated in WYY, including homologs of *salicylic acid-binding protein* (*SABP2*), *sucrose synthase 6* (*SUS6*), and an NADPH: *quinone oxidoreductase* (Supplementary Table S6).

### Transcription Factors Related to the Synthesis of Flavonoids

**Figure 5:**
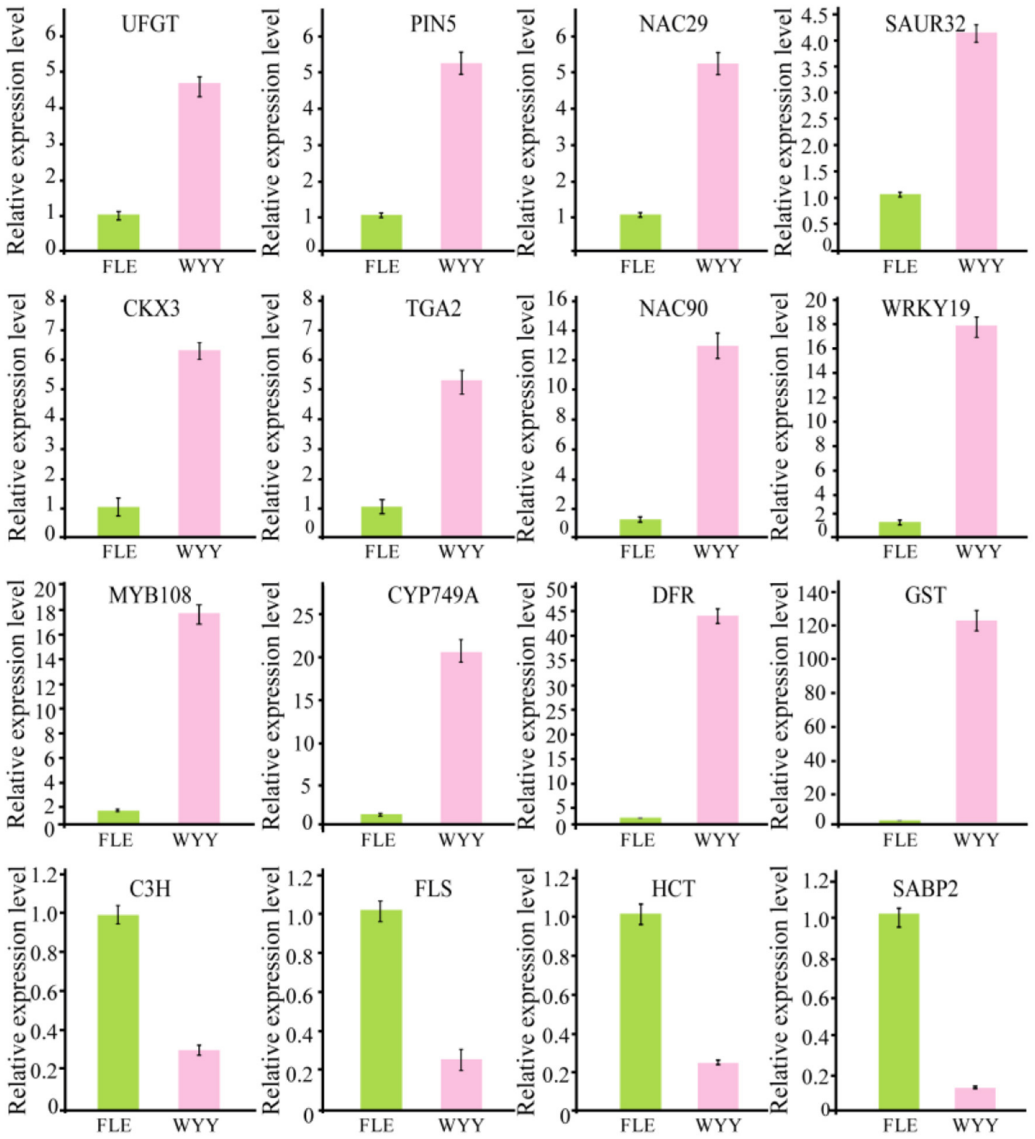
Validation of RNA-seq data by qRT-PCR.

Transcription factors play an important role in the regulation of flavonoid biosynthesis by regulating the expression level of structural genes. In our study, 17 TFs of 7 TF families were found. Among the TF families, MYB (3 unigenes) and B3 (2 unigenes) were the most prominent, followed by NAC (2 unigenes), WRKY (1 unigene), HD-ZIP (1 unigene), bZIP (1 unigene) and bHLH (1 unigene) (Supplementary Table S7). Almost all MYB DEGs belong to the R2R3 MYB family, which has been reported to participate in regulating anthocyanin synthesis in multiple species. Two MYB genes encoding *MYB75* and *MYB108* were identified, and transcription analysis showed that *MYB108* had a higher expression level in the red stems. We infer that these TF families play an important role in the structural gene regulation of WYY stem coloration. To verify the accuracy of RNA-seq data, 16 genes, including the candidate structural genes together with the key transcription factors, were selected and a quantitative real-time PCR (qRT-PCR) was performed. The results showed that the gene expression profiles were well consistent with the RNA-seq data, which further demonstrated the credibility of the data generated in our study (Figure 5, Supplementary Table S8).

### Comprehensive Analysis of Metabolome and Transcriptome

**Figure 6:**
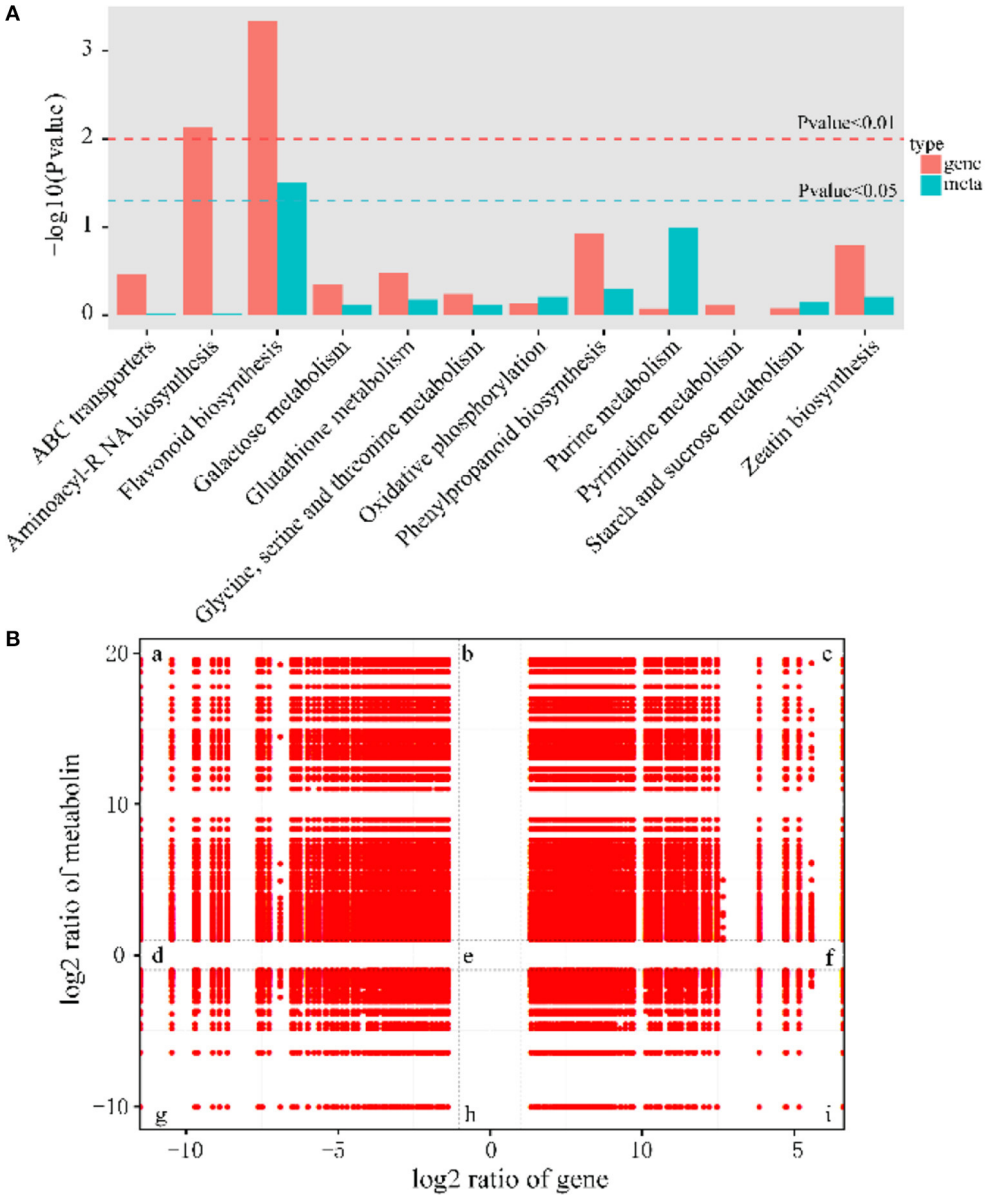
Significantly related KEGG pathways and nine quadrant diagrams. **(A)** Integrated analysis of KEGG pathways of metabolome and transcriptome. **(B)** Nine squares indicated nine responsive groups: (a) Transcriptionally down-regulated and metabolically up-regulated genes. (b) Transcriptionally unchanged and metabolically up-regulated genes. (c) Transcriptionally and metabolically up-regulated genes. (d) Transcriptionally down-regulated and metabolically unchanged genes. (e) Transcriptionally and metabolically unchanged genes. (f) Transcriptionally up-regulated and metabolically unchanged genes. (g) Transcriptionally and metabolically down-regulated genes. (h) Transcriptionally unchanged and metabolically down-regulated genes. (i) Transcriptionally up-regulated and metabolically down-regulated genes. The X axis represents log2 expression ratios of gene and Y-axis represents log2 expression ratios of metabolin.

To investigate the relationship of DEMs and DEGs involved in the same biological process (KEGG pathway), the co-expression analysis of metabolome and transcriptome was performed using Pearson's correlation coefficient. There were many pathways for simultaneous annotation of differential metabolites and differential genes. We picked genes and metabolic pathways with *p* < 0.05 for priority analysis, which can save the time of data screening and quickly find the pathways for subsequent analysis. These pathways are summarized in Figure 6A, and the results indicated that the most representative category was the flavonoid biosynthesis pathway.

To obtain the potential relationship between genes and metabolites, the FPKM of transcription level and metabolic level was calculated, and then screened according to the canonical correlation analysis (CCA), with the following parameters: |CC|>0.8 and CCP <0.05. Further analysis showed that 37% of the genes belonged to the congruent groups (group c and group g), and their expression trends were consistent at the transcriptional and metabolic levels, which may positively regulate the expression of metabolites. Meanwhile, 63% of the genes were located in the incongruent groups (group a and i), and the expression trends of genes and metabolites were opposite, which meant that genes and metabolites were negatively correlated (Figure 6B).

**Figure 7:**
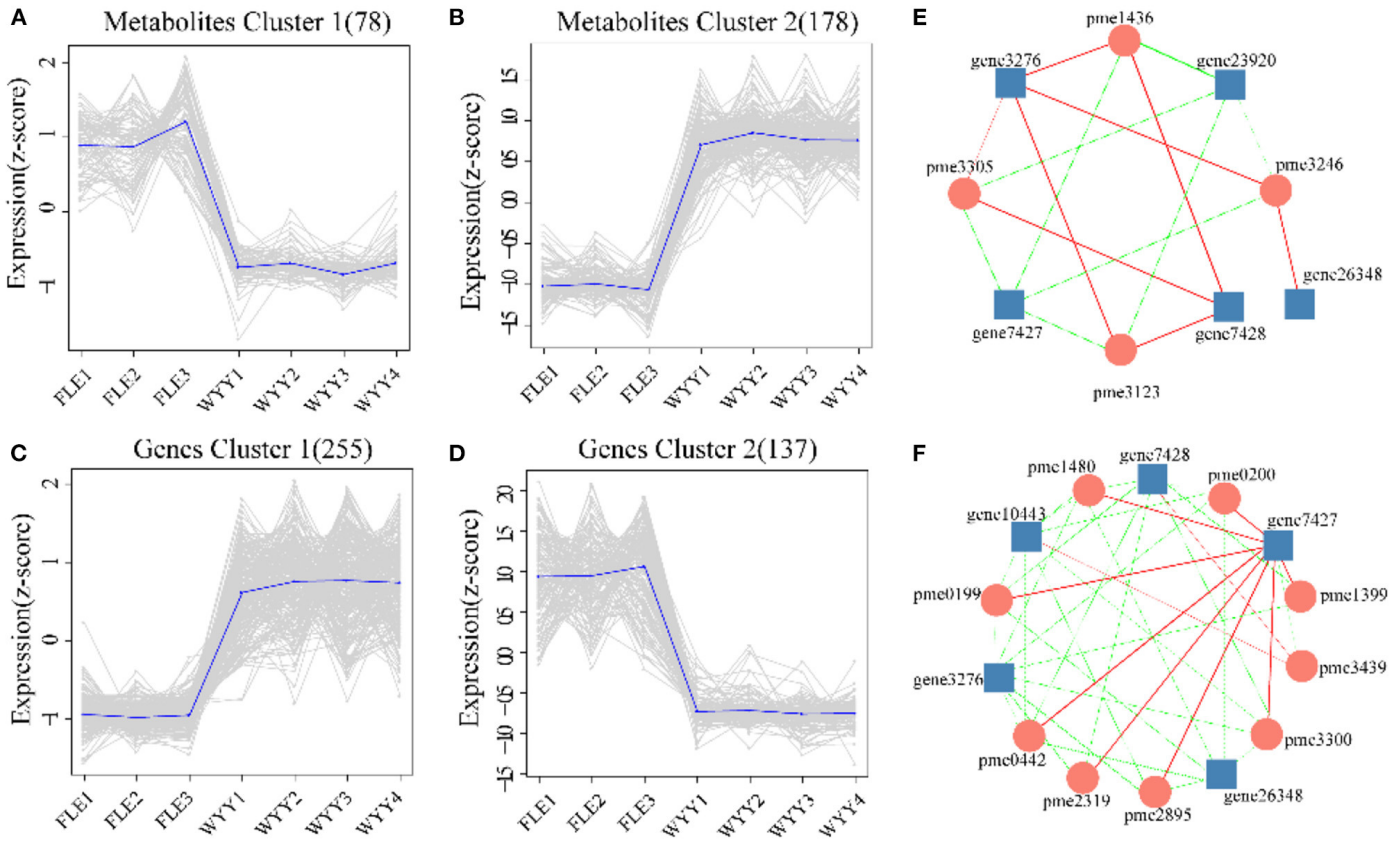
Trend analysis and the co-expression analysis of DEGs and DEMs. **(A,B)** K-means clustering analysis of the DEMs into two clusters according to their expression profile. **(C,D)** K-means clustering analysis of the DEGs into two clusters according to their expression profile. **(E)** Interaction network of DEGs and DEMs involved in phenylpropanoid biosynthesis. **(F)** Interaction network of DEGs and DEMs associated with flavonoids biosynthesis. Metabolites are shown in circles and gene IDs are shown in squares. Edges colored in “red” and “green” represent positive and negative correlations, respectively.

Next, we focused on metabolites and genes with consistent trends. All significantly differentially expressed metabolites and genes were classified by K-means method, and four clusters were identified (Figures 7A–D). Compared with Metabolites cluster 1 and Genes cluster 2, which showed an overall downward trend, Metabolites cluster 2 and Genes cluster 1 displayed an overall upward trend. Genes cluster 1 encompassed 255 genes, which were highly expressed, suggesting a positive correlation with red stem formation in WYY. Conversely, 137 genes in Genes cluster 2 were down-regulated, which might be negatively associated with the red coloration. KEGG analysis also revealed that flavonoid biosynthesis and phenylpropanoid biosynthesis were significantly enriched in Genes cluster 1.

### The Coexpression Analysis of DEGs and DEMs in Flavonoid Biosynthesis Pathway

The co-expression networks of DEGs and DEMs were mainly enriched in phenylpropanoid biosynthesis (Figure 7E) and flavonoids biosynthesis (Figure 7F). We found that p-Coumaric acid, p-Coumaryl alcohol, sinapyl alcohol, and coniferin were positively correlated with the transcription expression of *Pm008812* encoding HCT (hydroxycinnamoyl-CoA shikimate transferases), and *Pm003887, Pm003886*, both encoding C3H (cytochrome P450 98A2). In contrast, *Pm008809*, encoding HCT, and *Pm028093* (beta-glucosidase 12) were negatively related to these metabolites. The enzymes encoded by these genes catalyzed the conversion between p-coumaroyl CoA and p-coumaroyl shikimic/quinic acid, which would be critical for the synthesis of caffeoyl shikimic/quinic acids. C3H/HCT determines the flow of carbon sources in plants and plays an important role in the phenylpropanoids pathway. Whether p-coumarin-CoA forms H-lignin or G/S-lignin depends on the activity of C3H/HCT. The DEGs and DEMs, such as *CHS* (*Pm012349*), *C3H*(*Pm003886*), kaempferol and myricetin, quercetin, delphinidin were found to be involved in the biosynthesis of flavonoids.

## Discussion

**Figure 8:**
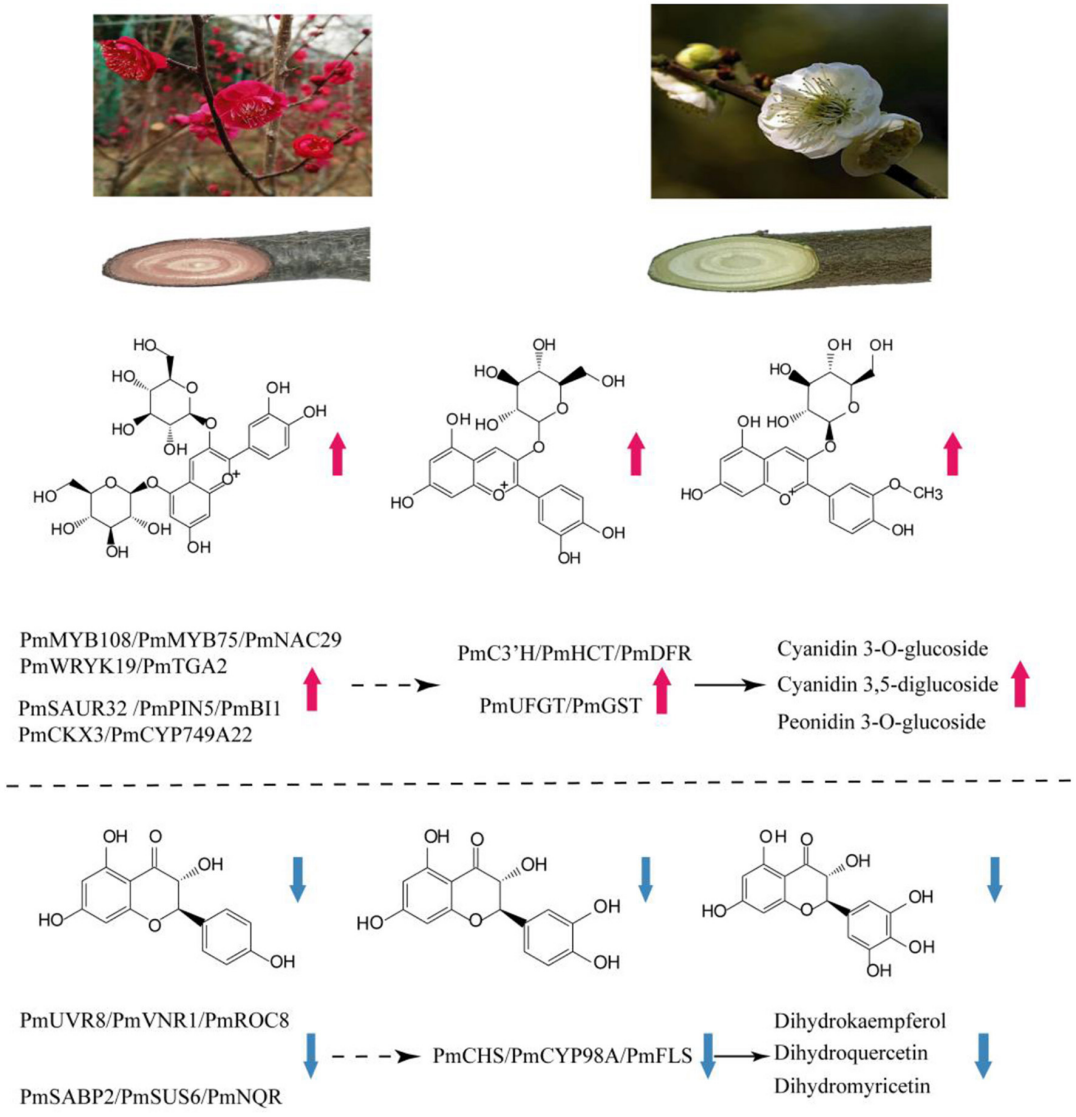
Summary of transcription-level regulation of the red stem formation in *P. mume*. Transcripts and metabolites show obvious differences in WYY and FLE. Total anthocyanins content and expression of *C3'H, HCT, DFR, UFGT, GST* genes are increased, resulting in preferential anthocyanin pathway in WYY. Moreover, the increased expression of *CHS, CYP98A and FLS* causes a higher proportion of flavonols in FLE. The solid black arrow indicates direct control; the dashed arrow indicates indirect regulation.

Deciphering the mechanism of plant coloration has always been a hot topic in ornamental horticulture. However, previous studies mainly focused on the leaves, flowers, fruits, seeds, epidermis and other organs or tissues of herbaceous plants, and there was no report on the formation mechanism of xylem color trait of woody plants. In this work, as an effort to reveal the underlying molecular mechanisms of the formation of different colors in stems of *P. mume*, a combined metabolome and transcriptome study was designed. Upon comparison of the differentially accumulated metabolites in the different cultivars, the contents of flavonoids and anthocyanins were the main reason for the difference in stem color. A hypothetical model for red stem formation in *P. mume* is summarized in Figure 8. Higher anthocyanin content was detected in WYY, while a very small amount of anthocyanins were accumulated in FLE, which was consistent with the HPLC results in other cultivars. Twenty-four anthocyanins were identified in WYY and the high accumulation levels of cyanidin 3,5-O-diglucoside, cyanidin 3-O-rutinoside, and peonidin 3-O-glucoside were considered to be the main components of red pigmentation. In addition, a large amount of procyanidin A1, quercetin, tricetin, and other important secondary metabolites were accumulated in the biosynthetic pathway of phenylpropanoid and flavonoids. It is worth noting that some anthocyanin compounds were accumulated only in WYY, in particular cyanidin glycosides and pelargonidin glycosides. This is in line with the previous studies, which found that red flowers and white flowers contained the same non-red flavonoids, and there was a significant positive correlation between red pigmentation and anthocyanin content in *P. mume* (Zhao et al., 2004). Ma et al. similarly found that cyanidin 3-O-glucoside, cyanidin 3,5-O-diglucoside, and peonidin 3-O-glucoside were the primary anthocyanins in pink petals, while no such substances were detected in *P. mume* with white petals (Ma et al., 2018). We also found that several anthocyanins were present simultaneously in WYY and FLE, which may be because, in addition to the amount and type of total anthocyanins, the intensity of anthocyanins were affected by a variety of complex factors, such as vacuolar pH, co-pigmentation, and metal chelation (Manetas, 2006). The same predominant anthocyanins were found in three different colored poinsettia bracts (Slatnar et al., 2013). In our study, quercetin and kaempferol were significantly accumulated in the flavonoid synthesis pathway, which is the upstream of the anthocyanin pathway. Low levels of epicatechin, which compete with anthocyanins for the same substrate, and high levels of flavonoids provide sufficient substrates for anthocyanin accumulation.

On the basis of GO and KEGG analysis of transcriptome data, we suggest that phenylpropanoid synthesis and flavonoid biosynthetic pathway may strongly influence the formation of red stems. The increased gene transcription levels in these pathways strongly support our metabolome results. Anthocyanin biosynthetic pathway has been found to regulate color formation in many plants, such as *Camellia sinensis* (Zhou et al., 2020), *Paeonia suffruticosa* (Gu et al., 2019), waterlily (Wu et al., 2016), and *Senecio cruentus* (Jin et al., 2016). Based on the expression levels and fold changes, some genes related to anthocyanin biosynthesis were exploited. In terms of the upstream genes, CHS, as a key enzyme affecting the accumulation of flavonoids, can catalyze the condensation of coumaric acid CoA into chalcone derivatives, which form the basic skeleton of downstream flavonoids. However, in our study, we found that a *CHS* homologous unigene was down-regulated in WYY and negatively related to the accumulation of kaempferol and myricetin, which may be affected by the feedback mechanism of the flavonoid pathway in plants. The transcriptome expression pattern revealed that compared with the flavonols biosynthesis, WYY preferentially flows to the anthocyanin pathway by up-regulating the expression of *DFR* and down-regulating one *FLS* gene, which can catalyze dihydroflavanols to produce leucocyanidin and flavonols, respectively. It has been reported that the metabolic balance in the flavonoid biosynthetic pathway is affected by the ratio of *FLS/DFR* (Gou et al., 2011). Here, one differentially expressed *DFR* homologous unigene was identified in WYY, and its expression level was 4-fold higher than that in FLE. Conversely, the *FLS* was highly expressed in FLE. Furthermore, correlation analysis between metabolites and transcripts showed that anthocyanin content was positively correlated with *DFR* and negatively correlated with *FLS*. The silencing of *McDFR* increased the accumulation of flavonols, whereas inactivation of *McFLS* elevated anthocyanin content in crabapple (Tian et al., 2015). In grape hyacinth, a highly expressed *FLS* along with a lowly expressed *DFR* lead to the fading of blue pigmentation (Lou et al., 2014). It provides a new perspective for breeders to cultivate new varieties of ornamental plants with novel colors.

On the other hand, DFR is a rate-limited enzyme, which can specifically catalyze one or more dihydroflavonols to produce the corresponding leucoanthocyanidins due to the different enzymatic sites in the conserved domain. In peanuts, DFR does not catalyze dihydrokaempferol and therefore fails to produce pelargonidin, resulting in lack of brick-red flowers (Johnson et al., 1999). Down-regulating endogenous *DFR* expression levels and over-expressing the *iris DFR*, which preferred dihydromyricetin, yielded a blue rose rich in delphinidin (Katsumoto et al., 2007). In our study, WYY was able to accumulate all three anthocyanins, indicating that DFR belonged to the non-specific DFR enzymes and can convert all types of dihydroflavonols. However, there were significant differences in the contents of different types of anthocyanins. Therefore, how *DFR* affect the formation of different colors needs further study.

Anthocyanins are easily degraded, so glycosylation is an indispensable part of the process of anthocyanin accumulation in plants. Under the catalysis of UFGT, the hydrophilicity and stability of aromatic rings were increased by adding sugar moieties to the 3-OH position of anthocyanin (Lo Piero, 2015). The *GST* catalyzed the formation of a relatively stable complex between glutathione and anthocyanin and transported it to vacuoles for storage (Mol et al., 1998; Grotewold, 2006). In apple, the activity of UFGT was positively correlated with the accumulation of anthocyanins (Ji et al., 2015). In our study, we detected that one *UFGT* gene and several *GST* genes were strongly up-regulated, possible contributing to the high accumulation of anthocyanins. This is consistent with findings in grape, which found that *UFGT* was highly expressed in red-skin grapes, but not in white cultivar tissues, and *UFGT* was up-regulated at veraison (Boss et al., 1996).

Glutathione s-transferase (GST) is a family of proteins with abundant physiological functions, which plays an important role in plant detoxification and secondary metabolism. There are two hypotheses for the mechanism of anthocyanin transport from endoplasmic reticulum to vacuoles: vesicle-trafficking model and transporter model (Liu et al., 2019). Both models infer that GST was significant for the efficiency of anthocyanin transport. Our results also suggest that *GST* may be indispensable for anthocyanin pigmentation. We detected three *GSTs* in all four WYY samples, which were almost undetectable in FLE samples, and a *multidrug and toxic efflux transporter protein* (*TT12*) was significantly up-regulated in WYY. The effect of GST on conjugation of glutathione and anthocyanins was first demonstrated in maize *bz2* mutant (Marrs et al., 1995). In petunia, *AN9*, which was regulated by transcriptional activator of anthocyanin pathway, performed a similar function to *bz2* and complemented the phenotype of *bz2* mutant (Alfenito et al., 1998). Based on research in peach, a small indel mutation in *Riant*, which encoded a GST, made the red petals to fade or even turn white (Cheng et al., 2015). *Arabidopsis* MATE family contained 56 genes, each of which performed different functions, and their transport substances were different. *AtTT12* is located in the tonoplast and function to transport proanthocyanidin precursors epicatechin-3-O-glucoside into the vacuole (Marinova et al., 2007). More recently, MATE2 from *Medicago truncatula*, homologous *to TT12*, was found to efficiently transport cyanidin 3-O-glucoside (Zhao et al., 2011). On the whole, high expression levels of anthocyanin-related genes including *DFR, UFGT, GST*, and *TT12*, may play key roles in the direction and distribution of flavonoids metabolic flux and the accumulation of anthocyanins in WYY.

Anthocyanin synthesis has specific synergies among structural genes and is regulated by a transcription complex formed by R2R3-MYB, bHLH, and WD40 transcription factors (MBW). The function of the MBW complex has been widely verified in plants, such as *Prunus persica* (Uematsu et al., 2014), *Lilium* (Yamagishi, 2010), and *Actinidia chinensis (Peng et al.*, *2019**)*. In peony, *PsMYB12* interacted with a bHLH and a WD40 protein in a regulatory complex that directly activated *PsCHS* expression (Gu et al., 2019). Furthermore, *LcR1MYB1* and *LcNAC13* acted together to antagonistically regulate anthocyanin biosynthesis during litchi fruit ripening (Jiang et al., 2019). We identified several members of these transcription factors. One unigene encoding *MYB108* exhibited high expression levels in WYY, and several members of this gene family had been reported to mediate flower color in plants (Takahashi et al., 2011; Zhu et al., 2015). However, *MYB75*, highly homologous to *A. thaliana MYB113*, was down-regulated. Besides, we also observed that a bHLH transcription factor was up-regulated, which was involved in tapetal cell development. However, no annotated gene related to WD40 was found in DEGs. Our recent studies showed that a petal color-associated QTL was located in the same area of *MYB108* on chromosome Pa4. Transcriptome data also indicated that *MYB108* was specially expressed in the red petals of WYY (Zhang et al., 2018). Furthermore, we found that the expression of some hormones and sugar-related proteins, such as *efflux carrier component 5* (*PIN5*), *auxin response protein SAUR32, brassinosteroid insensitive 1* (*BRI1*), and sucrose synthase 6, changed significantly in WYY. The production of anthocyanin in *O. linearis* callus cultures was regulated by the feedback of auxin concentration, which was inhibited at high concentrations and promoted at low concentrations (Meyer and Van Staden, 1995). Liu et al. (2014) showed that auxin increased the expression of *DFR* and *ANS* genes by stimulating TTG1-TT8-PAP1 complex, thereby regulating the accumulation of anthocyanins in red pap1-D *Arabidopsis* cells. It has been proved that sugar-phosphorylation interacting with related signal transduction can induce gene expression and anthocyanin accumulation in developing petunia flowers (Solfanelli et al., 2006). We hypothesize that these differentially expressed transcription factors can be candidate regulators of WYY anthocyanin biosynthesis, but their molecular and physiological functions are still unclear and need further study.

## Conclusion

In this research, we systematically studied the formation mechanism of red stem in WYY through multi-omics analysis. The high accumulation of metabolites in anthocyanin-related synthetic pathways, especially cyanidin glycoside and paeoniflorin glycoside, were considered to be the main sources of red pigmentation. The transcriptome and the correlation analyses revealed that differentially expressed structural genes and transcription factors, such as *FLS, DFR, UFGT, MYB75*, and *MYB108*, may contribute to modulating the formation of the red stems in WYY. Our results provide a reference for molecular mechanism of xylem color trait in *P. mume* and lay a theoretical foundation for cultivating new varieties.

## Materials and Methods

### Plant Materials

Two *P. mume* cultivars with different stem colors (‘Wuyuyu' with red stem and ‘Fei Lve' with green stem) were chosen for transcriptome and metabolome analyses. Both cultivars were grown in the same nursery of Beijing Forestry University. Stems are selected from three different individuals of each cultivar based on size, length, and degree of lignification. The samples were frozen in liquid nitrogen for subsequent transcriptome and metabolome analyses. Transcriptome sequencing was performed on three biological replicates, and metabolic profile analysis was performed on four biological replicates. The cultivars ‘Wuyuyu' and ‘Fei Lve' were abbreviated as WYY and FLE, respectively.

### Metabolomics

For metabolomic analysis, the metabolites were extracted, identified, and quantitatively analyzed by Biomarker Technologies (Beijing, China). In brief, the freeze-dried stem was crushed and 100 mg of powder was dissolved in 1.0 mL of 70% methanol solution overnight at 4°C. After centrifugation and filtration, the extracted solution was analyzed using an LC-ESI-MS/MS system (HPLC, Shim-pack UFLC SHIMADZU CBM30A system; MS, Applied Biosystems 6500 Q TRAP). Before the data analysis, a quality control sample, which was a mixture of sample extracts, was prepared to monitor the repeatability of the sample under the same detection method. Mass spectrometry data were processed by Analyst 1.6.1 software (AB Sciex). Principal component analysis (PCA) and Hierarchical cluster analysis (HCA) were used to evaluate the metabolic differences between different samples. Metabolites were considered to be differentially accumulated if the fold change was≥2 and variable importance in project (VIP) was≥1. Enrichment analysis of differential metabolites was compared to the KEGG database and clustered by using cluster Profiler in R.

### Anthocyanin Content Measurement

Four cultivars were used to determine the content of anthocyanins, including ‘Wuyuyu', ‘Fenhong Zhusha', ‘Fei Lve' and ‘Zaohua Lve'. First, 0.25 g of powder sample per cultivar was added into 5 mL of methanol, distilled water, formic acid, and trifluoroacetic acid (70:27:2:1, v/v/v/v), and extracted at 4°C for 24 h. After centrifugation, the supernatants were filtered by a medium-speed filter paper and a 0.22 μm syringe filter (Millipore, Bedford, MA, USA) before subjecting it to HPLC-MS/MS analysis. The mobile phase was 2% formic acid in water (phase A) and 0.1% formic acid acetonitrile solution (phase B) at a flow rate of 0.6 mL/min. The linear gradient of phase B was as follows: 0 min 5% B, 20 min 28% B, 30 min 60 B%, 45 min 28% B, 45–60 min 5% B. The UV-visible light detector wavelength was set at 520 nm for detecting anthocyanins. The mass spectrometry analysis conditions were as follows: electrospray ionization (ESI^+^); ion trap analyzer; scan mode: total ion scanning; scanning range (m/z):100–1,000; capillary voltage: 4,000 V; sprayer pressure: 35 psi; dry gas: N2; dry temperature: 350°C. For preparation of the standard solution, we accurately weighed the cyanidin 3,5-O-diglycoside and diluted it to several different concentrations. The quantitative analysis was carried out according to the procedures described by Sun et al. (2009). Each sample was repeated three times under the same conditions.

### Transcriptomics

Total RNA of all stem samples was extracted using a RNAprep Pure Plant Kit (DP432, Tiangen, China). The quality and purity of RNA were evaluated by 1% agarose gel and NanoDrop 2000 (Thermo fisher Scientific, USA). Qualified RNA samples were sent to Biomarker Technology for cDNA library construction and sequencing. At least four biological repeats were designed per cultivar. Eight cDNA libraries were constructed and sequenced on an Illumina HiSeq 2500 platform. After removing the adapter and low-quality sequencing data, the obtained high-quality clean reads were aligned to the *P. mume* genome sequence using HISAT2, and then assembled and quantified using StringTie (Pertea et al., 2015). For functional annotation, all assembled unigenes were aligned to the public database by using BLASTX (Altschul et al., 1997), including Nr, Pfam, KOG/COG, Swiss-Prot, and KEGG. The differentially expressed genes (DEGs) were detected with DEGseq (Wang et al., 2010), and fold change≥2 and false discovery rate (FDR) <0.01 were used as threshold in the detection process. GOseq R package and KOBAS software were used for GO enrichment and KEGG analysis of the DEGs, respectively.

### Integrative Metabolome and Transcriptome Analysis

Pearson correlation coefficient was used to screen the correlation between metabolites and genes, with correlation coefficients> 0.8 and *P* < 0.05 as the selection criteria. The co-expression analysis between differential expression metabolites (DEMs) and differential expression genes (DEGs) in phenylpropanoid and flavonoids biosynthesis were visualized in Cytoscape (v3.3.0).

### Expression Pattern Analysis

The transcript levels of 16 genes were subjected to qRT-PCR. The specific gene primers were designed by Integrated DNA Technologies (https://sg.idtdna.com) based on reference sequences from *P. mume* genome. All reactions were conducted using the TB Green® Premix Ex Taq™ II (TaKaRa, Beijing, China) on the PikoReal Real–Time PCR System (Thermo Fisher Scientific). The 2-delta-delta Ct method was used to calculate gene expression, and *Protein phosphatase 2A* (*Pm006362*) was selected as an internal reference control. All analytical procedures were carried out with three biological replicates.

## Data Availability Statement

The datasets presented in this study can be found in online repositories. The names of the repository/repositories and accession number(s) can be found below: https://ngdc.cncb.ac.cn/search/?dbId=gsa&q=CRA006277.

## Author Contributions

LQ and TZ conceived and drafted the manuscript. LQ, TZ, WL, XZ, and PL analyzed the data. JW and TC provided of plant resources. TZ and QZ contributed to the conception of the study and finalized the manuscript. All authors read and approved the final manuscript.

## Funding

The research was supported by the National Natural Science Foundation of China (Nos. 32071816 and 31800595), and the Special Fund for Beijing Common Construction Project.

## Conflict of Interest

The authors declare that the research was conducted in the absence of any commercial or financial relationships that could be construed as a potential conflictof interest.

## Publisher's Note

All claims expressed in this article are solely those of the authors and do not necessarily represent those of their affiliated organizations, or those of the publisher, the editors and the reviewers. Any product that may be evaluated in this article, or claim that may be made by its manufacturer, is not guaranteed or endorsed by the publisher.

## Supplementary Material

Supplementary Figure S1 Principal component analysis of the metabolome samples.

Supplementary Figure S2 Heat map of the metabolic profiling.

Supplementary Figure S3 OPLS-DA model plots.

Supplementary Figure S4 KEGG Pathway classification and functional enrichment of DEMs.

Supplementary Figure S5 Pearson correlation between eight libraries.

Supplementary Figure S6 GO functional analysis and classification of DEGs.

Supplementary Table S1 All metabolite information identified by widely-targeted metabolomics.

Supplementary Table S2 Differentially expressed metabolites between WYY and FLE.

Supplementary Table S3 Statistics of RNA-seq reads and genome alignment in 8 samples.

Supplementary Table S4 Differentially expressed genes between WYY and FLE.

Supplementary Table S5 DEGs related to flavonoid transport.

Supplementary Table S6 DEGs related to sugar and hormones metabolism.

Supplementary Table S7 Differentially expressed transcription factors.

Supplementary Table S8 Primers used for qRT-PCR.
