# Supplementary figures and images for: Integration of Transcriptome and Metabolome Reveals the Formation Mechanism of Red Stem in Prunus mume
Source: Front Plant Sci. 2022 May 6;13:884883. doi: 10.3389/fpls.2022.884883 (PMC9120947; doi:10.3389/fpls.2022.884883)

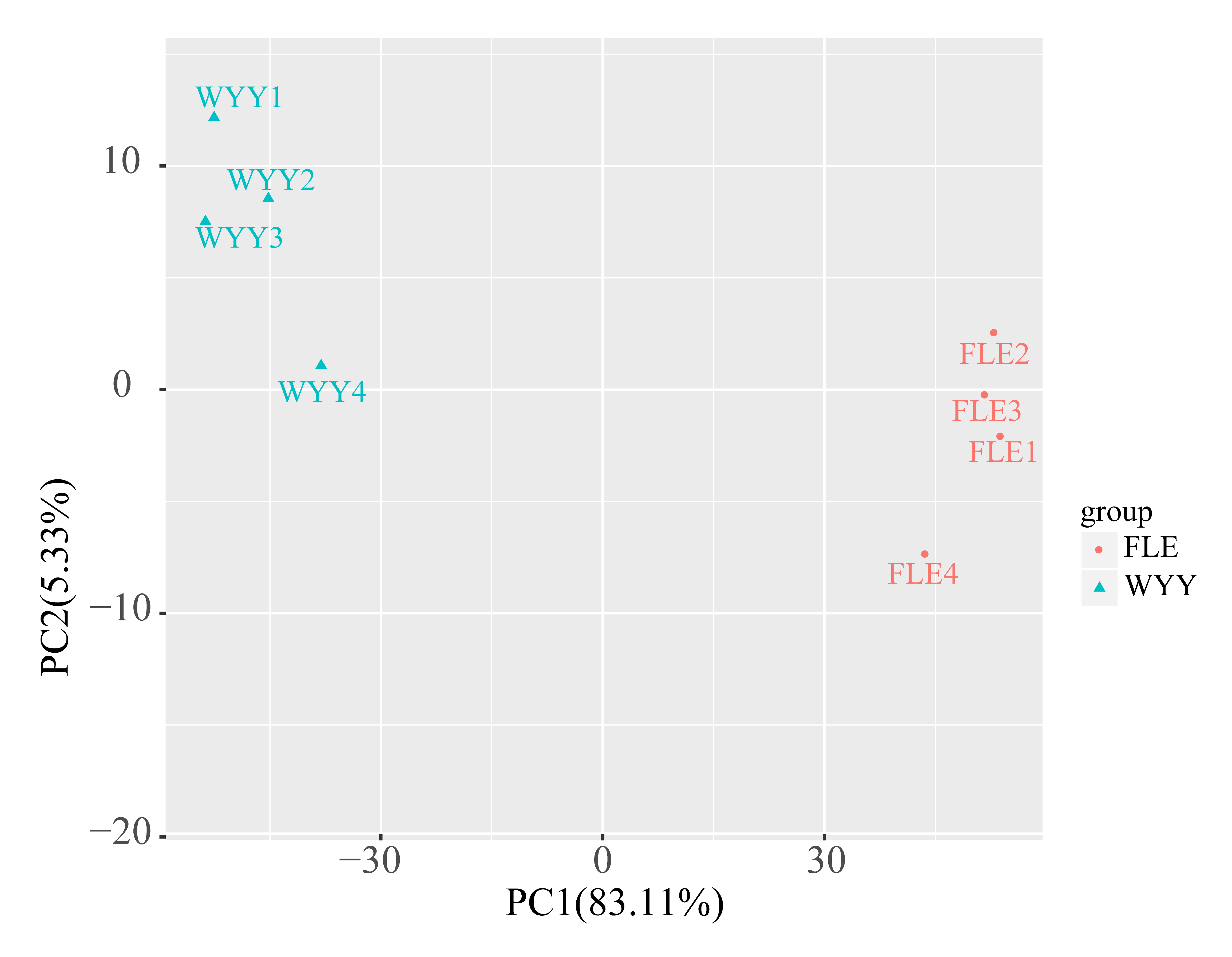

Supplement: Supplementary Figure S1 — Principal component analysis of the metabolome samples. [file Image_1.tif]

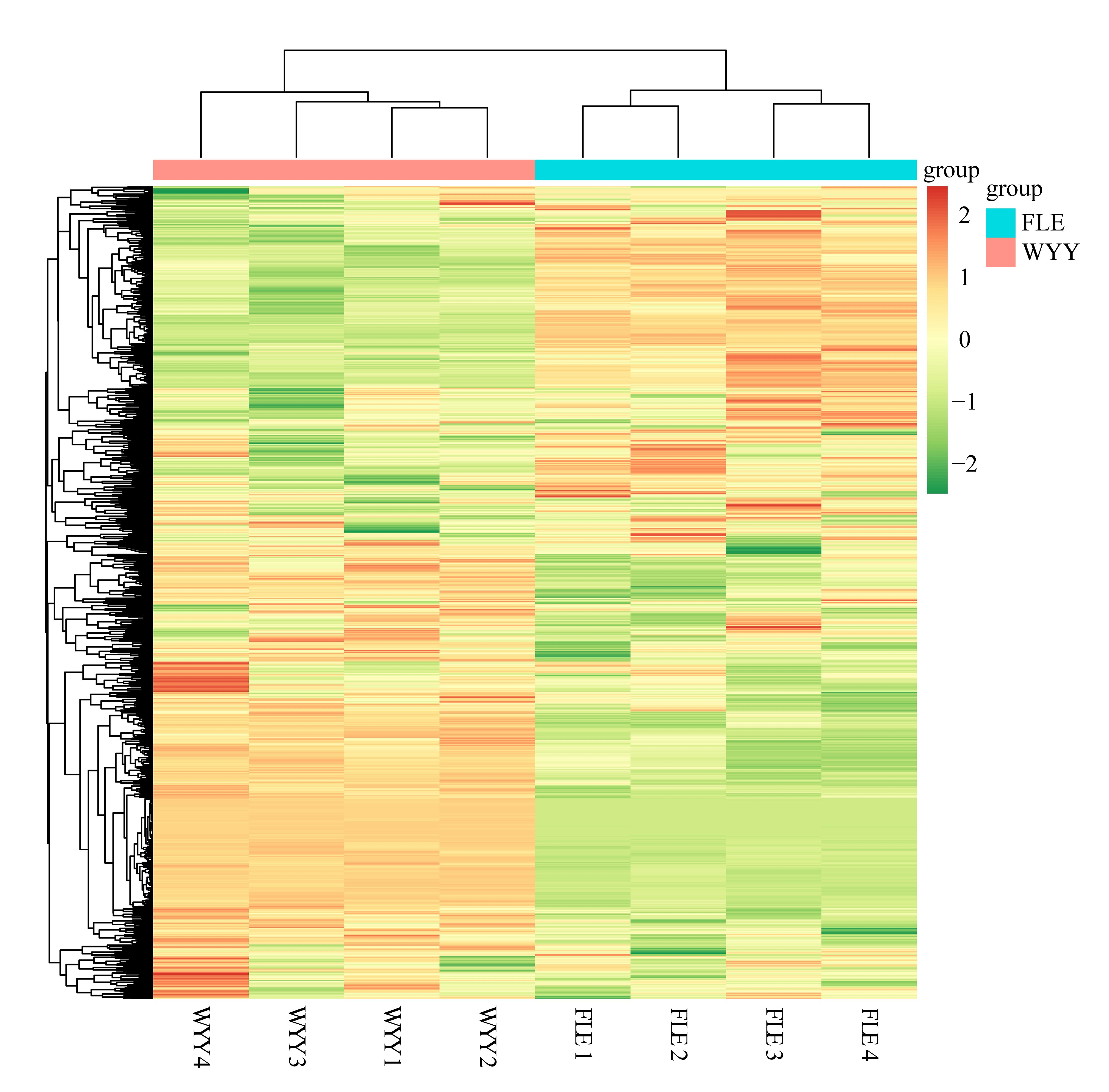

Supplement: Supplementary Figure S2 — Heat map of the metabolic profiling. [file Image_2.tif]

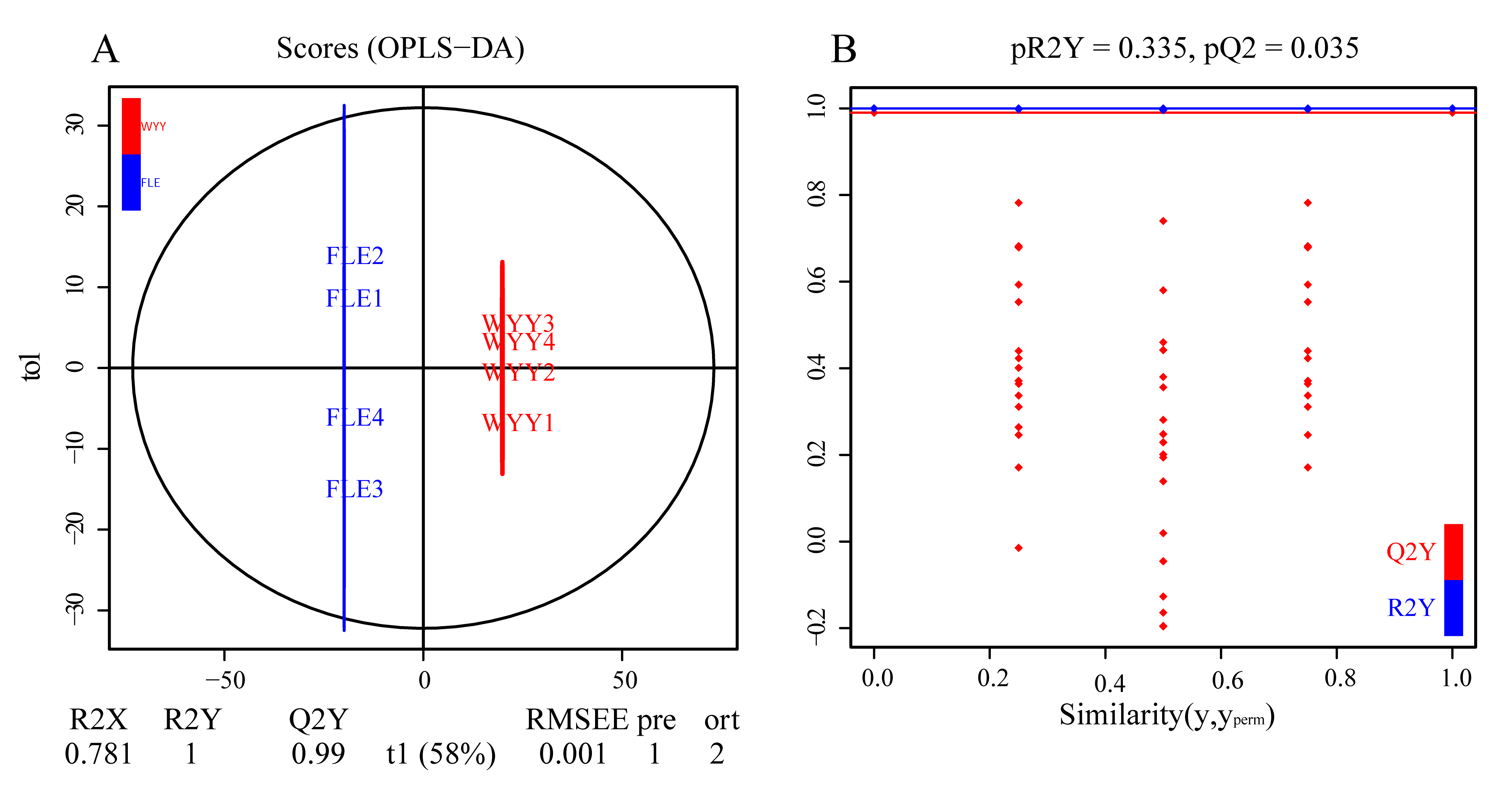

Supplement: Supplementary Figure S3 — OPLS-DA model plots. [file Image_3.tif]

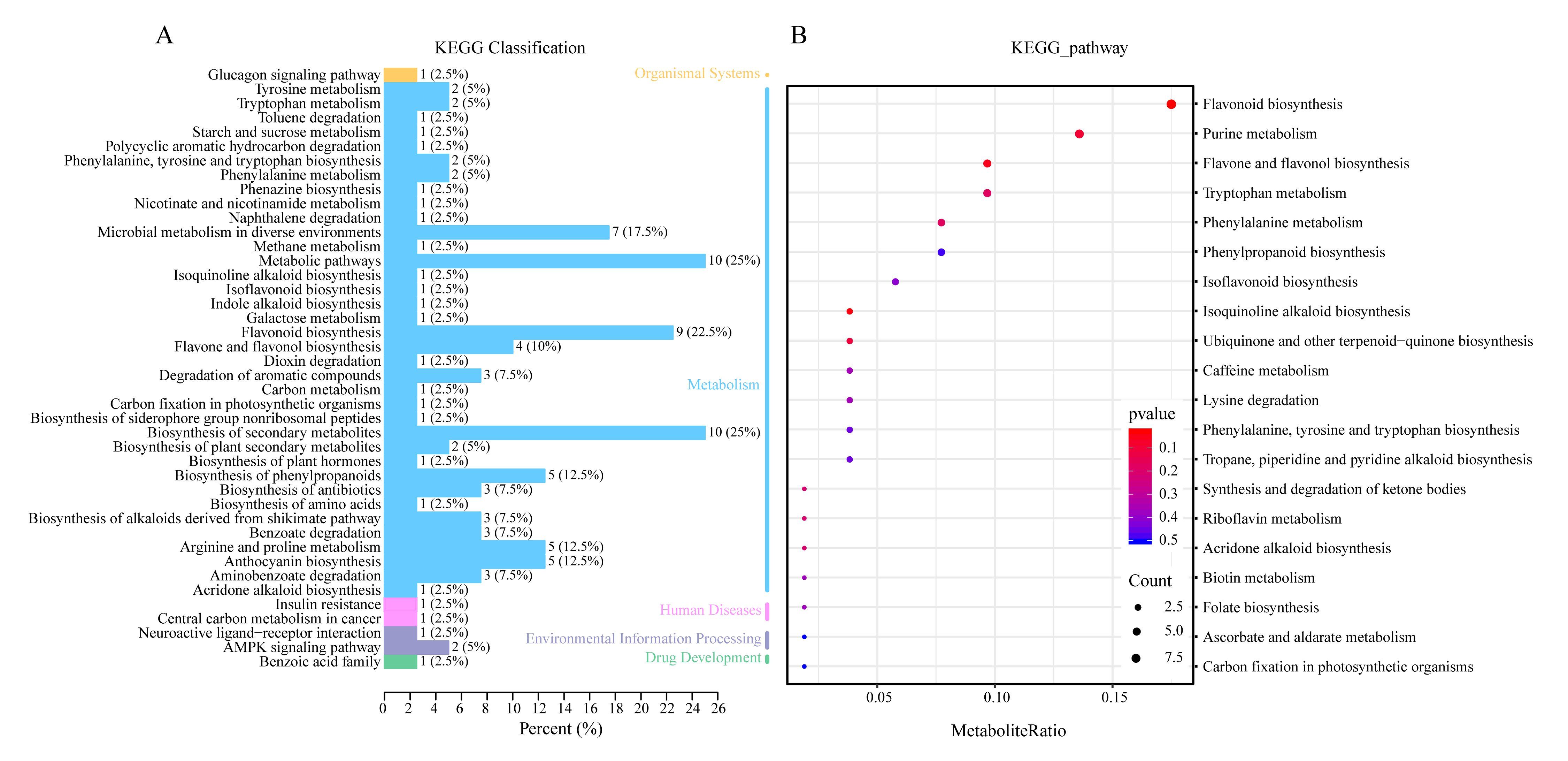

Supplement: Supplementary Figure S4 — KEGG Pathway classification and functional enrichment of DEMs. [file Image_4.tif]

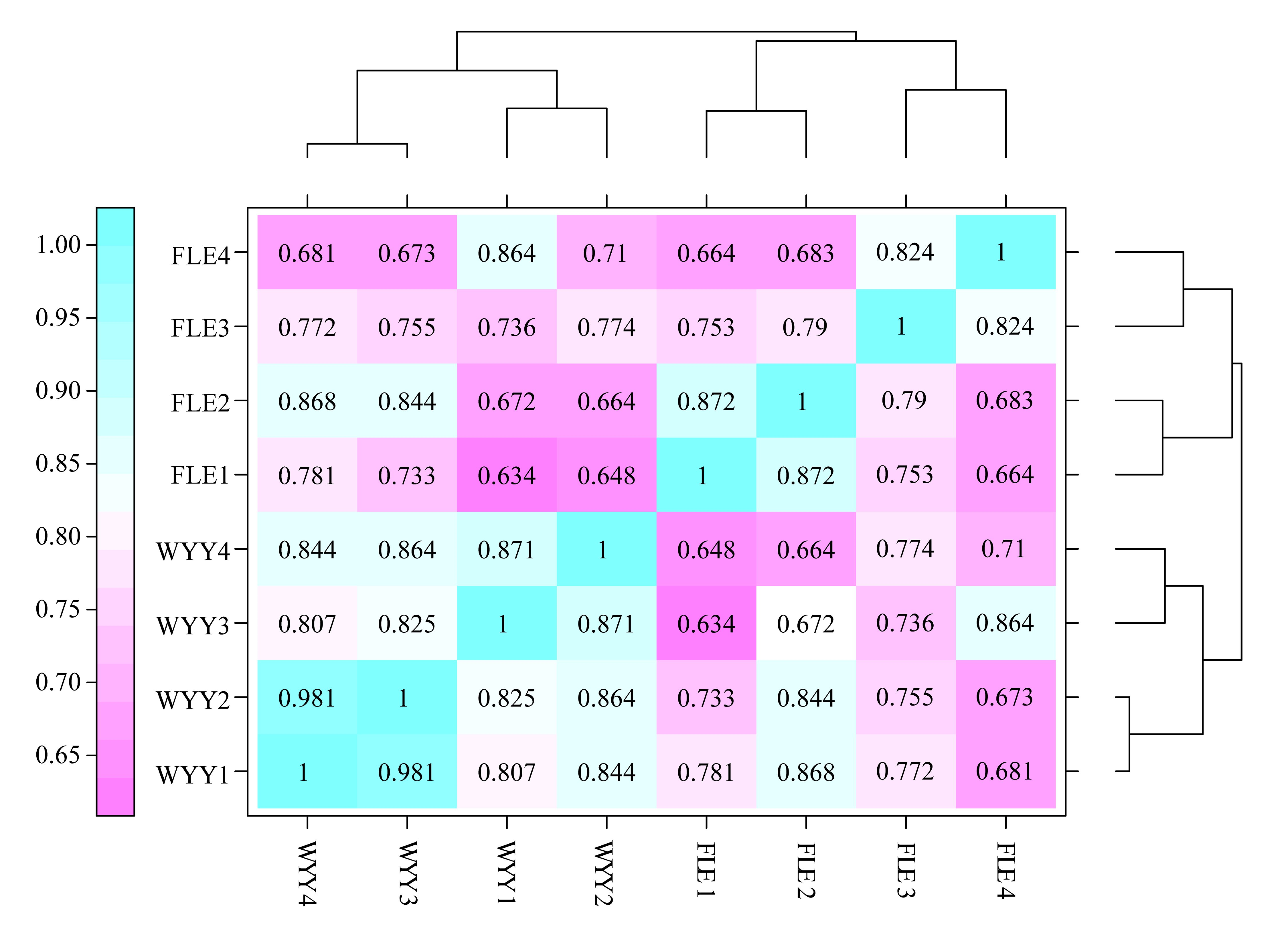

Supplement: Supplementary Figure S5 — Pearson correlation between eight libraries. [file Image_5.tif]

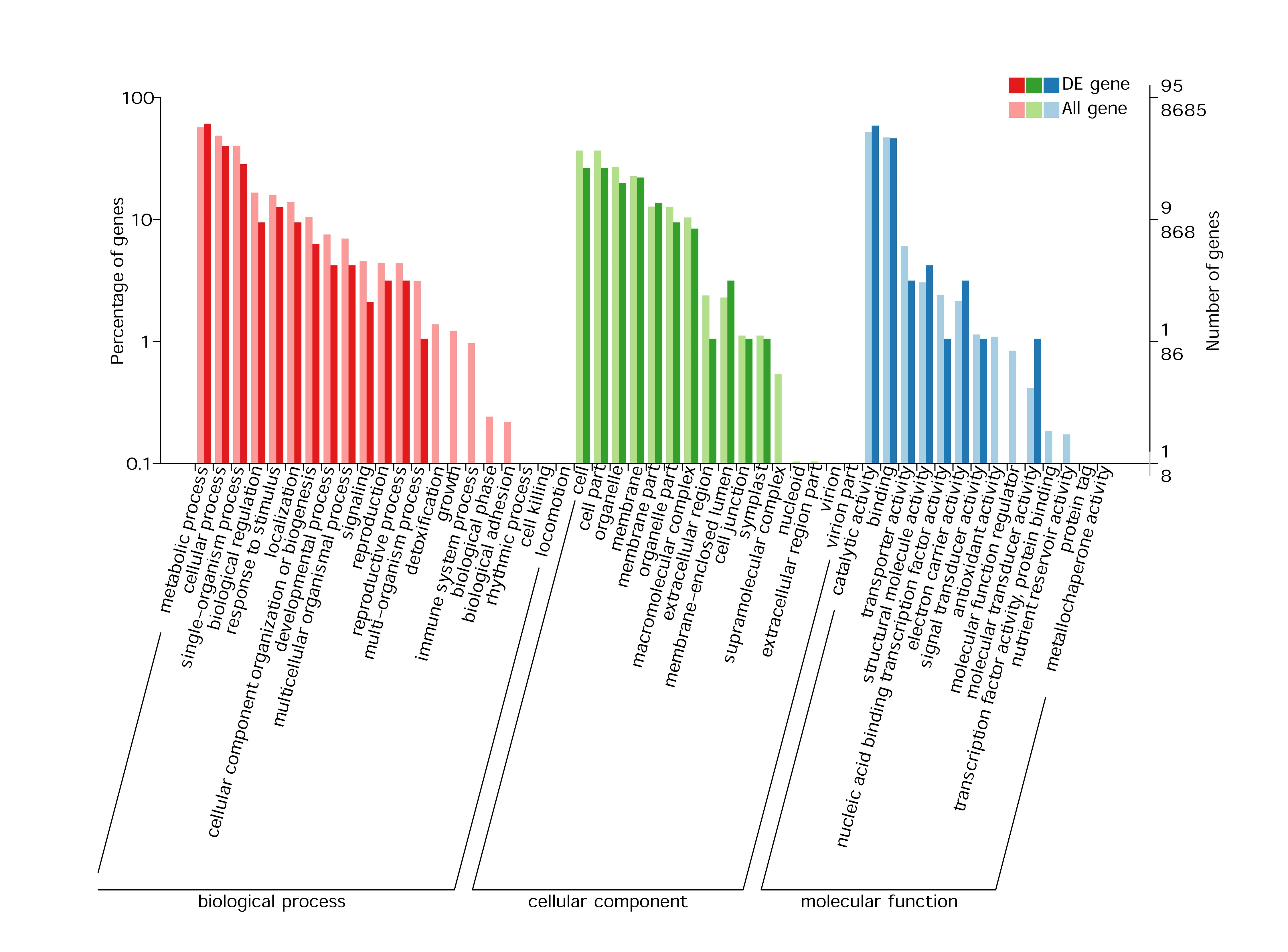

Supplement: Supplementary Figure S6 — GO functional analysis and classification of DEGs. [file Image_6.tif]
